# Rich Intercultural Music Engagement Enhances Cultural Understanding: The Impact of Learning a Musical Instrument Outside of One’s Lived Experience

**DOI:** 10.3390/ijerph20031919

**Published:** 2023-01-20

**Authors:** Marjorie G. Li, Kirk N. Olsen, Jane W. Davidson, William Forde Thompson

**Affiliations:** 1 School of Psychological Sciences, Macquarie University, Macquarie Park, NSW 2109, Australia; 2 Australian Institute of Health Innovation, Macquarie University, Macquarie Park, NSW 2109, Australia; 3 Faculty of Fine Arts and Music, University of Melbourne, Southbank, VIC 3006, Australia; 4 Faculty of Society and Design, Bond University, Robina, QLD 4226, Australia

**Keywords:** intercultural music engagement, embodiment, affordance, cultural empathy, tolerance, social connection, attitudes, racial bias, non-Western music training

## Abstract

Rich intercultural music engagement (RIME) is an embodied form of engagement whereby individuals immerse themselves in foreign musical practice, for example, by learning a traditional instrument from that culture. The present investigation evaluated whether RIME with Chinese or Middle Eastern music can nurture intercultural understanding. White Australian participants were randomly assigned to one of two plucked-string groups: Chinese *pipa* (*n* = 29) or Middle Eastern *oud* (*n* = 29). Before and after the RIME intervention, participants completed measures of ethnocultural empathy, tolerance, social connectedness, explicit and implicit attitudes towards ethnocultural groups, and open-ended questions about their experience. Following RIME, White Australian participants reported a significant increase in ethnocultural empathy, tolerance, feelings of social connection, and improved explicit and implicit attitudes towards Chinese and Middle Eastern people. However, these benefits differed between groups. Participants who learned Chinese *pipa* reported reduced bias and increased social connectedness towards Chinese people, but not towards Middle Eastern people. Conversely, participants who learned Middle Eastern *oud* reported a significant increase in social connectedness towards Middle Eastern people, but not towards Chinese people. This is the first experimental evidence that participatory RIME is an effective tool for understanding a culture other than one’s own, with the added potential to reduce cultural bias.

## 1. Introduction

Intercultural tensions are a global threat, ranging from anti-Muslim sentiment due to fear of terrorism, to Chinese people being blamed and vilified for the COVID-19 pandemic, to violence against First Nations communities and people of colour. Underlying such tensions is a lack of cultural empathy and implicit biases that have developed through prolonged exposure to biased media and pervasive social attitudes. For example, Muslims are frequently portrayed as aggressive, violent, and threatening in the public media [1]. Therefore, strategies are needed to promote intercultural understanding, defined as a state of empathising, appreciating, and respecting others from different cultural backgrounds. 

A significant challenge in identifying such strategies is that cultural differences are vast and may include differences in religious beliefs, norms for dress and communication, gender roles, and degrees of individualism or collectivism. We contend, however, that embodied engagement with a traditional cultural practice may inspire cultural tolerance and open-mindedness. Music represents an ideal introduction to another culture because it does not need to conflict with long-standing belief systems. As such, intercultural music engagement (IME) provides a simple and benign activity through which one can learn to understand the traditions of another culture. 

Although IME can be achieved by merely listening to music (e.g., [2]), we predict that a more substantial level of engagement may be needed for a genuine change in cultural attitudes to take place. We define rich intercultural music engagement (RIME) as a form of intercultural music engagement that includes an embodied, performative component, designed to provide a deeper experience than mere listening, and that results in a nuanced, embodied experience of the culture. RIME brings participants inside another culture, as opposed to observing a cultural practice at “a distance” or at a tokenistic level more akin to music listening rather than embodied music engagement.

What features of music and underlying mechanisms might account for such an impact? According to the Therapeutic Music Capacities Model, music acts like a combination therapy by triggering numerous mechanisms that ultimately have implications for cognitive, psychosocial, behavioural, and motor function [3]. Such outcomes occur because music encompasses several design features or capacities: it is simultaneously engaging, emotional, physical, personal, social, and persuasive, and affords synchronisation of action with others. In therapeutic contexts, music can be strategically employed to address therapeutic goals such as the enhancement of movement, timing, memory, communication, and self-awareness. 

**Figure 1:**
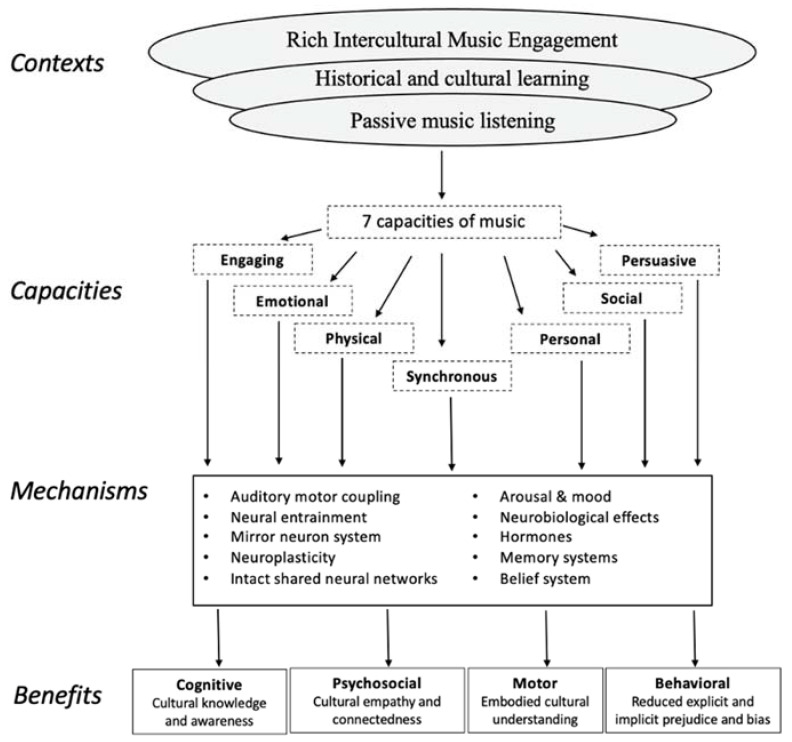
The Intercultural Music Capacities Model (IMCM). The IMCM illustrates the contexts, capacities, mechanisms, and outcomes of engaging with music from another culture. Rich intercultural music engagement is proposed as the most effective means of achieving outcomes related to intercultural understanding.

It is reasonable to assume that engaging in music from another culture activates the same set of physical and psychological mechanisms, but results in outcomes that also provide cultural understanding. As music is a powerful carrier of cultural information, music engagement can provide an effective means of attaining cultural understanding [4,5]. Figure 1 illustrates an adapted version of the Therapeutic Music Capacities Model, referred to as the Intercultural Music Capacities Model (IMCM). The figure shows the various contexts of music engagement, the potential capacities of that engagement, the mechanisms that are triggered, and the potential intercultural outcomes. Rich intercultural music engagement involves an active, participatory, embodied music experience, and is likely to be one of the most effective means of achieving intercultural understanding. 

Ethnographic evidence supports the premise of Figure 1 and suggests that Westerners who have an immersive, embodied experience of a non-Western musical culture feel they gain a strong intercultural understanding [6,7]. However, experimental studies are needed to assess the reliability and validity of such anecdotal claims. One experimental study conducted by Vuoskoski [2] suggested that listening to music from non-Western cultures might slightly reduce implicit bias for the people of those cultures, but this effect was marginal and only evident for a subset of participants who scored high on empathy. Another cross-cultural longitudinal study by Neto et al. [8] revealed that a 6-month singing program reduced racial prejudice immediately following the program and two years later. However, strong conclusions could not be drawn owing to the small sample size available for the retest. Moreover, the design of the study makes it difficult to know whether music engagement was responsible for these effects. For example, using songs as learning stimuli makes it difficult to disentangle the effects of lyrical content with the effects of music. Therefore, our study used instrumental music (no lyrics) as the intervention.

The present study was designed to examine the effects of RIME on intercultural understanding for White Australians. In this study, White Australians refer to people living in Australia who do not identify as First Nations or a person of colour. RIME was operationalised as *the acquisition of rich contextual knowledge of a non-Western musical culture through learning to play a non-Western musical instrument from that culture*. Two plucked-string instruments, Chinese *pipa* and Middle Eastern *oud,* were used to represent two ethnocultural groups. By learning the culture-specific musical instruments, participants were given the opportunity to acquire intercultural understanding at an embodied, experiential level. It was hypothesised that participating in RIME with Chinese or Middle Eastern music should increase White Australians’ ethnocultural empathy and tolerance (H1), feelings of connection (H2), and positive attitudes (H3), while reducing negative implicit biases towards different ethnocultural groups (H4). We also surmised there may be differences in the impact of RIME for the two cultural groups, perhaps reflecting the extent to which biases for Chinese and Middle Eastern people are entrenched or recently acquired.

## 2. Materials and Method

### 2.1. Participants

An *a priori* power analysis suggested a minimum of 54 participants to achieve a medium effect size of 0.25, α = 0.05, power [1-β] = 0.95 [9] and similar previous research has recruited 61 participants [2]. In this study, 58 participants were recruited: 38 Macquarie University psychology students in exchange for course credit points and AUD20 cash; 20 participants external to Macquarie University in exchange of AUD20 cash and the opportunity (1 in 5 chance) to win an AUD50 Amazon gift voucher. 

The final analysis consisted of 26 females and 32 males ranging in age from 17 to 67 years (*M* = 29.78, *SD* = 15.91). A total of 29 participants were randomly assigned to the Chinese *pipa* group, 17 female and 12 male ranging in age from 17 to 67 years (*M* = 28.86, *SD* = 15.33); and 29 participants were assigned to the Middle Eastern *oud* group, 9 female and 20 male ranging in age from 18 to 67 (*M* = 30.68, *SD* = 16.69). Overall, 58.6% of participants reported some form of previous musical training. 

The inclusion criteria were: White Australians who (a) had no prior experience playing a Middle Eastern *oud* or a Chinese *pipa*; (b) did not speak Arabic or Chinese; and (c) did not have any close relationships with people from these regions for six months or longer. Ethics approval was granted by the Macquarie University Human Research Ethics Committee, Reference No: 52021955024986. 

### 2.2. RIME Intervention Programs

RIME intervention programs were developed by two trained specialists, one from each of the two non-Western musical cultures, and the first author who has expertise in Western music, the two non-Western musical cultures, and in teaching musical instruments. Each RIME program contained two learning phases. Part A presented a two-hour pre-recorded lecture on musical culture, including four modules: (a) an introduction to the cultural history and social context related to the designated instrument (~30 min); (b) an introduction to a wide range of performance practices in different ritual, operatic, narrative, and folk ensembles and diverse musical traditions, including three exemplar performance clips (~30 min); (c) an overview of the instrument, e.g., conventional and alternative tunings, notation, and basic playing techniques, including four exemplar performance clips (~30 min); and (d) a module that demonstrated a White student learning to play a folk song from the trained specialist (~30 min). This final module consisted of learning the basics in instrumental playing techniques, learning from observation, learning from imitation, and then playing together. Part B was a two-hour hands-on instrumental learning session where the participant sat face-to-face with the first author to learn a folk song. The content from the fourth module in Part A was revisited and used as learning material in Part B. This learning sequence aligns with the active ingredients from the IMCM framework (see Figure 1) and ensures participants were fully engaged in every aspect of embodied learning.

To minimise any effects related to the two different qualified experts used in the recorded RIME sessions for the Middle Eastern *oud* and Chinese *pipa* groups, the two experts followed the same procedure (demonstration, imitation), thus interacting with participants in a manner that was highly consistent with one another. In addition, all participants, regardless of the cultural group, were exposed to the same structured lesson outlines. Thus, it is unlikely that the observed differences can be explained merely by differences in the approach or demeanour of the two experts. Furthermore, employing the same expert for the recorded RIME sessions would have introduced a different kind of confound related to the differential level of familiarity and comfort of the expert with the two non-Western instruments.

### 2.3. Measures

#### 2.3.1. Musical Engagement Style

This variable was measured to explore the individual’s level of music engagement in cognitive and emotional regulation, music production, social connection, physical exercise, and dance. The Musical Engagement Scale [10] was used as a 24-item questionnaire, each item rated on a 6-point Likert scale (0 = “Not at all/Not applicable to me” to 5 = “Strongly agree”). Total score ranged from 0 to 120. High scores indicated a high level of music engagement. 

#### 2.3.2. Mutual Intercultural Relations in Plural Societies (MIRIPS) Questionnaires

The MIRIPS questionnaires [11] contain a series of measures and four subscales were used to assess: security (SEC), perceived discrimination (PD), tolerance/prejudice (TOL), and attitudes towards ethnocultural groups (ATT). SEC and PD are rated on a 5-point Likert scale (1 = “Strongly disagree” to 5 = “Strongly agree”). SEC is a 13-item scale that assesses the sense of security from perspectives of multiculturalism. A high score reflects a high sense of security, which may promote greater acceptance of others and positive attitudes towards other ethnocultural groups in a multicultural society. Cronbach’s alpha for SEC in MIRIPS studies ranges between 0.47 and 0.63 [12]. PD is a 5-item scale that measures the sense of fear one may experience in a multicultural society. The high score reflects a high perceived discrimination or threat, which may prevent acceptance of others and be negatively associated with attitudes towards other ethnocultural groups. Cronbach’s alpha for PD in MIRIPS studies ranges between 0.73 and 0.86 [12]. 

The TOL subscale of the MIRIPS is an 11-item scale that taps into ethnic tolerance and attitudes on social equality. Each item is rated on a 5-point Likert scale (1 = “Strongly disagree” to 5 = “Strongly agree”). Total score ranges from 11 to 55 with a high score indicating high tolerance towards ethnic others. Cronbach’s alpha for TOL in MIRIPS studies ranges from 0.52 to 0.71 [12]. The ATT subscale uses a sliding scale from 0–100 to measure explicit attitudes (via ratings of favouritism) towards Chinese people, Middle Eastern people, and Australian people. The higher the score, the more positive attitude towards each group. A score of 50 indicates neither unfavourable nor favourable towards each group.

#### 2.3.3. Ethnocultural Empathy

Ethnocultural empathy was measured using the Scale of Ethnocultural Empathy (SEE) [13]. The SEE is a 31-item questionnaire that measures explicit empathy towards people of different racial and ethnic backgrounds. Each item is rated on a 6-point Likert scale (1 = “Strongly disagree” to 6 = “Strongly agree”, no neutral option). The total score ranges from 31 to 186, with high scores indicating a high level of ethnocultural empathy. The SEE taps into four sub-domains [13]: (a) empathic feeling and expression; (b) empathic perspective taking; (c) acceptance of cultural differences; and (d) empathic awareness. The SEE yields high internal consistency (Cronbach’s alpha = 0.91) and test–retest reliability (*r* = 0.91).

#### 2.3.4. Social Connectedness

**Figure 2:**
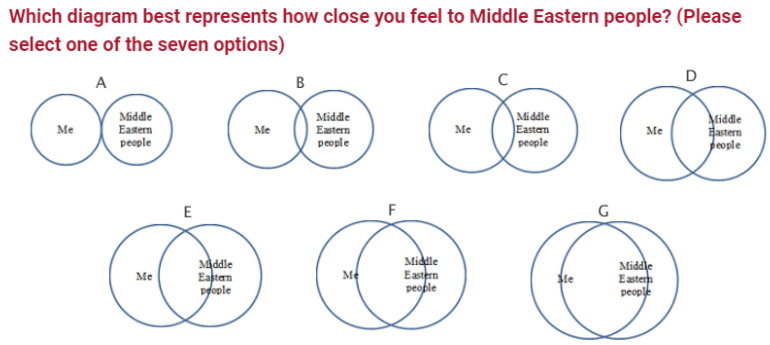
An Example of a Modified Version of the Inclusion of Other in the Self (IOS) Scale.

This variable was measured using a modified version of the Inclusion of Other in the Self (IOS) Scale [14]. The IOS is a single-item, pictorial measure tapping directly into people’s perceived relationship of closeness. The scale is rated on a 7-point Likert-style scale and consists of seven Venn diagrams, progressively showing a degree of self–other overlapping (see Figure 2). The IOS scale has sound psychometric properties with Cronbach’s alpha of 0.93 and a test–retest reliability of *r* = 0.83. In this study, three sets of IOS scales were implemented to measure social connectedness before and after the RIME intervention: Me vs. Middle Eastern (IOS_M), Me vs. Chinese people (IOS_C), Me vs. Others (IOS_O) (“Me” denotes “White Australian”).

#### 2.3.5. Implicit Attitudes

Implicit prejudice/biases against people from different racial and ethnic backgrounds reflect internalised, entrenched attitudes that can persist in the absence of conscious awareness. The Implicit Association Test (IAT) [15] measures mean latency differences in incompatible/compatible sorting tasks (e.g., sorting target faces and positive/negative attributes into categories). *D*-scores are used to measure the strength and direction of the implicit association between targets (e.g., faces) and categories (positive/negative attributes). Australian and Middle Eastern face images used in the IAT were sourced from searching public websites. Chinese face images were sourced from the Tsinghua facial expression database [16]. All face images were pre-tested and verified as White Australian, Chinese, and Middle Eastern people. 

Three-set IATs were developed to test implicit association between ingroup/outgroup racial face images and positive/negative words, in the same way as a previous study investigating the impact of music listening on implicit affiliation [2]. There were three sets of IATs designed to measure implicit preference: Australian people versus Chinese people, Australian people versus Middle Eastern people, and Chinese people versus Middle Eastern people. Each IAT contained seven blocks of trials and four permutations were used to counterbalance left/right starting positions of targets and categories (see Table 1 for an example). Block 1, Block 2, and Block 5 required participants to sort Australian or Chinese faces using the letters “E” and “I” on the keyboard. The latency of their response time was not measured. Block 3 and Block 4 required participants to respond to the compatible pairs (White Australian faces with positive words; Chinese faces with negative words), while Block 6 and Block 7 required participants to respond to the incompatible pairs (White Australian faces with negative words; Chinese faces with positive words). The response times obtained from compatible pairing trials (Block 3 and 4) and incompatible pairing trials (Block 6 and 7) were used to calculate *D*-scores. The presentation order of the three sets of IATs was further randomised (six permutations) to minimise any order effects. 

The Qualtrics implementation of the IATs utilised the survey software IAT developed by Carpenter et al. [17]. The survey software is open source with free download and technical details available from the Open Science Framework https://osf.io/ntd97/ (accessed on 17 February 2021). The survey-software IAT was empirically validated against Millisecond’s Inquisit, a reaction-time software. The validation result found them comparable: similar reliability (split-half estimate 0.80 and 0.72, alpha 0.83 and 0.84), no difference in *D*-score results, and high correlations [17]. 

#### 2.3.6. Post-Experiment Feedback Survey

A short post-experiment survey consisting of five 5-Likert-style items (rated from 1 to 5) and two open-ended questions was implemented as a manipulation check. Participants were asked to consider their experience of the RIME and answer whether (1) it challenged me; (2) it gave me a sense of achievement; (3) it helped me understand [Chinese/Middle Eastern] culture; (4) it made me feel more connected to [Chinese/Middle Eastern] people; and (5) it made me feel more connected with others (all people in the world). The open-ended items were worded: (1) please list any other benefits you have received from learning a [Chinese *pipa*/Middle Eastern *oud*]; and (2) do you have further thoughts/feelings that you would like to share with us?

### 2.4. Procedure

**Figure 3:**
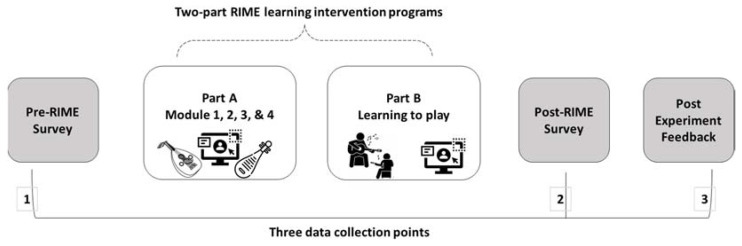
Schematic Representation of the RIME Design.

The survey components of this study were conducted online using Qualtrics. As indicated in the schematic diagram in Figure 3, participants were invited to take part through an email which included the Participant Information and Consent document and link to the survey. Participants then completed the online consent form before completing the pre-RIME survey for around 30 min. This survey consisted of demographic questions and the measures: MES, SEC, PD, TOL, ATTs, SEE, IOSs, and the IAT blocks. On completion of the survey, participants were *randomly* assigned to either the Chinese *pipa* or Middle Eastern *oud* group. Participants were contacted to visit the campus and attend the Part A two-hour lecture-style learning program. Following the learning session, participants were contacted again to visit the campus and attend the Part B two-hour hands-on instrumental playing session. After completing the learning programs, participants completed the post-RIME survey (around 25 min), which consisted of the following measures: SEE, TOL, ATTs, IOSs, and the IAT blocks. Afterwards, participants were invited to complete the optional post-experiment survey. 

### 2.5. Test of Assumptions

One statistical outlier with a *z*-score of −3.35 was detected using the *z*-score cut-off criterion of ±3.29 [18]. However, given the mean rating corresponding to this outlier was only 0.09 rating scale units lower than the next highest score on the TOL scale of 1–5, and the fact that it was the only outlier in a total of 116 TOL responses (58 participants x pre- and post-RIME responses), we chose not to transform or delete the outlier. The assumption of homogeneity of variance was not violated (Levene’s statistic *p*-value > 0.05). The assumption of homogeneity of covariances was not violated (Box’s Test *p*-value > 0.05). Shapiro–Wilk’s test revealed moderate violations of normality. No corrective steps were taken, given that the analysis of variance (ANOVA) method is robust to such violations if there are no outliers, and each condition has more than 20 participants and relatively equal participant numbers.

### 2.6. Test of Possible Covariates

A series of independent sample *t*-tests were conducted to compare individual factors between the two learning groups on age, musical engagement, sense of security, and perceived discrimination. The results revealed no significant differences between the two groups (*p*-values > 0.05). Therefore, these factors were not required as covariates in statistical models evaluating each hypothesis. 

## 3. Results

### 3.1. Ethnocultural Empathy (H1a)

The effect of the RIME intervention on ethnocultural empathy was examined using a 2 × 2 mixed-design ANOVA with learning group (Chinese *pipa*, Middle Eastern *oud*) as the between subjects factor and measurement time (pre-RIME intervention, post-RIME intervention) as the repeated measures factor. Mean ratings calculated from the 31-item SEE were used for this analysis. There was a significant main effect of measurement time for ethnocultural empathy, which significantly increased between pre-RIME (*M =* 4.59, *SD =* 0.54) and post-RIME intervention measurement times (*M =* 4.73, *SD =* 0.57), *F*(1, 56) = 14.27, *p* < 0.001, *η^2^_p_* = 0.20. There was no learning group x measurement time interaction (*p*-value > 0.05), meaning that the effect of RIME on ethnocultural empathy did not vary as a function of the learning group. These findings support H1 that participants trained on a Middle Eastern or Chinese instrument will experience an increase in ethnocultural empathy after exposure to the RIME intervention. A follow up analysis indicated that empathic awareness at baseline was lower for males (*n* = 32) than for females (*n* = 26), but increased significantly more following RIME for males than females, *F*(1, 56) = 5.87, *p* = 0.019, *η^2^_p_* = 0.10.

Further analysis of the SEE subdomains suggested that the effect of RIME on ethnocultural empathy was driven by improvements in empathic feelings and expression, empathic perspective taking, and empathic awareness. With both learning groups combined, empathic feeling and expression significantly increased between pre-RIME (*M =* 4.79, *SD =* 0.61) and post-RIME intervention measurement times (*M =* 4.90, *SD =* 0.68), *F*(1, 56) = 7.46, *p* = 0.008, *η^2^_p_* = 0.12. Empathic perspective taking significantly increased between pre-RIME (*M =* 3.58, *SD =* 0.80) and post-RIME intervention measurement times (*M =* 3.73, *SD =* 0.87), *F*(1, 56) = 4.08, *p* = 0.048, *η^2^_p_* = 0.07. Finally, empathic awareness significantly increased between pre-RIME (*M =* 4.81, *SD =* 0.90) and post-RIME intervention measurement times (*M =* 5.07, *SD =* 0.85), *F*(1, 56) = 12.05, *p* = 0.001, *η^2^_p_* = 0.18.

### 3.2. Tolerance (H1b)

The effect of the RIME intervention on tolerance was also examined using a 2 × 2 mixed-design ANOVA. Mean ratings calculated from the 11-item TOL subscale of the MIRIPS were used for this analysis. There was a significant main effect of measurement time, with a significant increase in tolerance between pre-RIME (*M =* 4.54, *SD =* 0.42) and post-RIME intervention (*M =* 4.64, *SD =* 0.33), *F*(1, 56) = 7.18, *p* = 0.010, *η^2^_p_* = 0.11. There was no learning group x measurement time interaction (*p*-value > 0.05), meaning that the effect of RIME on tolerance did not vary as a function of learning group. These findings support H1 that participants trained on a Middle Eastern or Chinese instrument experienced an increase in tolerance level towards ethnic groups after exposure to the RIME intervention.

### 3.3. Social Connectedness (H2)

The effect of the RIME intervention on social connectedness was examined by analysing a modified version of the IOS scale. Each learning group rated the magnitude of their social connectedness towards Chinese people, Middle Eastern people, and “Others” before and after the RIME intervention. It was hypothesised that participants in the Chinese *pipa* learning group would show a significant increase in social connectedness towards Chinese people after the RIME intervention, whereas participants in the Middle Eastern *oud* learning group would show a significant increase in social connectedness towards Middle Eastern people after RIME. 

**Figure 4:**
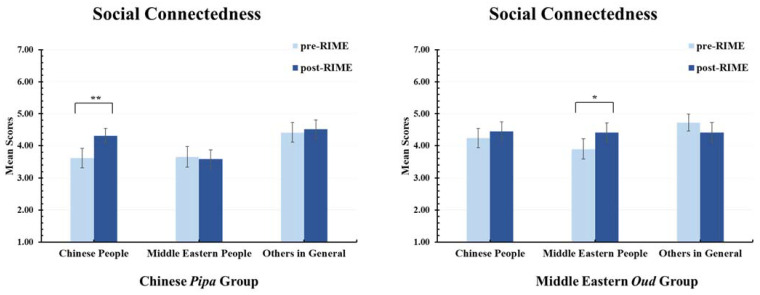
Social Connectedness Before and After the RIME Intervention. Left panel: the Chinese *pipa* group showed a significant increase in social connectedness towards Chinese people, but not towards Middle Eastern people or others; Right panel: the Middle Eastern *oud* group showed a significant increase in social connectedness towards Middle Eastern people, but not towards Chinese people or others. * *p* < 0.05. ** *p* < 0.01. Error bars report standard error of the mean.

As can be seen in the left panel of Figure 4, for participants in the Chinese *pipa* group, there was a significant increase in mean ratings of social connectedness towards Chinese people between pre- and post-RIME measurement times, *t* (28) = 2.83, *p* = 0.009, *d* = 0.53, 95% CI [0.13, 0.99]. There was no significant change in social connectedness towards Middle Eastern participants, *t* (28) = −0.47, *p* = 0.646, *d* = −0.09, 95% CI [−0.45, 0.28] or to other people in general, *t* (28) = 0.46, *p* = 0.648, *d* = 0.09, 95% CI [−0.28, 0.45]. Thus, H2 was supported for the Chinese *pipa* group. 

As can be seen in the right panel of Figure 4, for participants in the Middle Eastern *oud* group, there was a significant increase in mean ratings of social connectedness towards Middle Eastern people between pre- and post-RIME measurement times, *t* (28) = 2.48, *p* = 0.019, *d* = 0.46, 95% CI [0.07, 0.84]. There was no significant change in social connectedness towards Chinese participants, *t* (28) = 1.14, *p* = 0.264, *d* = 0.21, 95% CI [−0.16, 0.58], or to other people in general, *t* (28) = −1.27, *p* = 0.213, *d* = −0.24, 95% CI [−0.60, 0.14]. Thus, H2 was supported for the Middle Eastern *oud* group. Overall, the findings show that learning a musical instrument from another culture within the RIME intervention increased social connectedness towards that same culture. 

### 3.4. Explicit Attitudes towards Ethnocultural Groups (H3)

The effect of the RIME intervention on explicit attitudes towards ethnocultural groups was examined by analysing the ATT subscale of the MIRIPS. For each learning group, explicit attitudes towards Chinese people, Middle Eastern people, and Australian people were measured. The higher the score, the more positive attitude towards each group via ratings of favouritism. If the RIME intervention increases positive attitudes towards another cultural group, then we would expect a significant increase in positive attitudes towards Chinese people from participants in the Chinese *pipa* learning group, and similarly for positive attitudes towards Middle Eastern people from the Middle Eastern *oud* learning group. 

**Figure 5:**
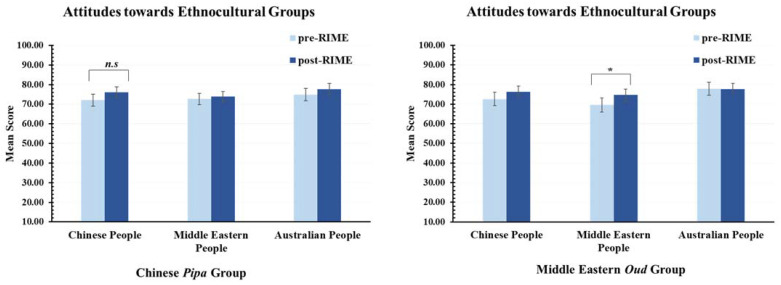
Explicit Attitudes towards Ethnocultural Groups Before and After the RIME Intervention. Left panel: the Chinese *pipa* group showed a non-significant increase in attitudes towards Chinese, Middle Eastern, or Australian people; Right panel: the Middle Eastern *oud* group showed a significant increase in attitudes towards Middle Eastern people, not Chinese or Australian people. * *p* < 0.05. *n.s.* = no significant difference. Error bars report standard error of the mean.

As can be seen in the left panel of Figure 5, for participants in the Chinese *pipa* group, paired sample *t*-tests (two-tailed) revealed no significant increase in explicit positive attitudes towards Chinese people, *t* (28) = 1.98, *p* = 0.058, *d* = 0.37, 95% CI [−0.01, 0.74]. There was also no significant change in explicit attitudes towards Middle Eastern people, *t* (28) = 0.60, *p* = 0.552, *d* = 0.11, 95% CI [−0.25, 0.48] or Australian people, *t* (28) = 1.27, *p* = 0.234, *d* = 0.23, 95% CI [−0.15, 0.59]. Thus, H3 was not supported for the Chinese *pipa* group. 

As can be seen in the right panel of Figure 5, for participants in the Middle Eastern *oud* group, there was a significant increase in explicit positive attitudes towards Middle Eastern people, *t* (28) = 2.13, *p* = 0.042, *d* = 0.40, 95% CI [0.01, 0.77]. There was no significant change in attitudes towards Chinese people, *t* (28) = 1.43, *p* = 0.165, *d* = 0.27, 95% CI [−0.11, 0.63] or Australian people, *t* (28) = −0.07, *p* = 0.949, *d* = −0.01, 95% CI [−0.38, 0.35]. Thus, H3 was supported for the Middle Eastern *oud* group. In summary, these findings show that learning a musical instrument from another culture within the RIME intervention increased explicit positive attitudes towards people of that culture for participants in the Middle Eastern *oud* learning group. RIME did not have a significant effect on explicit positive attitudes for participants in the Chinese *pipa* learning group.

### 3.5. Implicit Attitudes towards Ethnocultural Groups (H4)

Implicit attitudes towards ethnocultural groups were measured to address H4 by first calculating *D*-scores from responses according to the IAT score algorithm [19,20]. A series of one-sample *t*-tests analysed *D*-scores against zero (representing no implicit bias) to determine the direction and magnitude of each learning group’s implicit bias against Chinese, Middle Eastern people both pre- and post-RIME intervention. The IAT measures implicit bias through associated preference with targets, for example, Target A and Target B. Positive *D*-scores indicate a preference towards Target A and thus a negative bias against Target B. Negative *D*-scores indicate a preference towards Target B, and thus a negative bias against Target A. Following the criteria outlined by [2,21], a *D*-score between 0 and ±0.15 indicates neutral or little preference, a score between ±0.15 and ±0.35 indicates a slight preference, a score between ±0.35 and ±0.65 indicates a moderate preference, and a score greater than ±0.65 indicates a strong preference. 

**Figure 6:**
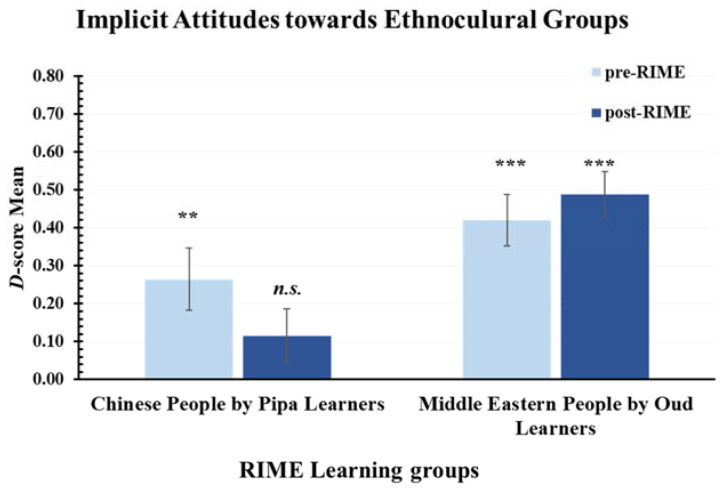
Implicit Attitudes Against Chinese and Middle Eastern People as Measured by the IAT. The panel on the left indicated that Chinese *pipa* learners showed a reduction in bias against Chinese people, from a slight bias pre-RIME to effectively no bias post-RIME; the panel on the right indicated that Middle Eastern *oud* learners showed no reduction in bias against Middle Eastern people, i.e., bias against Middle Eastern people were significant both pre-RIME and post-RIME. ** *p* < 0.01. *** *p* < 0.001. *n.s.* = no significant difference. Error bars report standard error of the mean.

As can be seen in Figure 6, participants in the Chinese *pipa* group exhibited implicit bias against Chinese people before the intervention (*M_D-score_* = 0.26, *SD* = 0.44) that was significantly higher than a neutral preference/*D*-score of zero (*p* = 0.003). After the intervention (*M_D-score_* = 0.11, *SD* = 0.38), this bias was reduced to a level that was not significantly different from a neutral preference/*D*-score of zero (*p* = 0.119). Thus, for participants in the Chinese *pipa* group, the RIME intervention reduced a significant bias against Chinese people to no significant bias against Chinese people. This result supported H4 for the Chinese *pipa* group.

Figure 6 also shows that participants in the Middle Eastern *oud* group exhibited a moderate to strong implicit bias against Middle Eastern people both before (*M_D-score_* = 0.42, *SD* = 0.36) and after the RIME intervention (*M_D-score_* = 0.49, *SD* = 0.32) that was significantly greater than a neutral preference/*D*-score of zero (*p*-values < 0.001). Thus, for participants in the Middle Eastern *oud* group, the RIME intervention did not significantly reduce any implicit bias against Middle Eastern people. This result did not support H4 for the Middle Eastern *oud* group, perhaps reflecting the entrenched nature of biases against Middle Eastern people. 

In summary, the results provided partial support for H4. Participants in the Chinese *pipa* group reduced implicit bias against Chinese people significantly but not towards Middle Eastern people. Participants in the Middle Eastern *oud* group did not reduce the implicit bias against Middle Eastern and Chinese people, and bias remained moderately strong. Thus, the RIME program only had the predicted effect of reducing implicit bias for those who learned the Chinese *pipa.*

### 3.6. Post-Experiment Feedback Survey

Forty-one participants provided feedback on five-item questions. Responses shown in Table 2 provided additional support for the main hypotheses, where participants were asked five questions on a 5-point Likert scale.

Thirty-two participants provided responses on one or both open-ended questions. Descriptions of felt experience towards the intercultural music engagement study were captured around four themes (e.g., enjoyable engagement, better understanding others’ music and culture, and people), supported by key constructs (e.g., intriguing; fun; interesting; amazing; open mind; respect; awareness). The reflections from participants exemplified how RIME made participants feel empathetic towards people associated with the musical culture, and how RIME helped them realise that cultural bias against other culture and people could happen unconsciously. 

## 4. Discussion

This study investigated the impact of rich intercultural music engagement (RIME) on cultural understanding and empathy through a two-part, four-hour intervention program comprising non-Western music training. The White Australian participants exhibited significant improvement in five key aspects of intercultural understanding after the intervention. First, ethnocultural empathy towards people of different racial and ethnic backgrounds was significantly increased following the RIME intervention. This increase was reflected by improvements in empathic awareness, as well as improvements in empathic perspective taking, empathic feelings, and empathic expression. Such benefits contrast with results of previous investigations involving non-musical interventions (e.g., [22]) and confirm that RIME provides an especially beneficial strategy for enhancing intercultural understanding. In addition, the main effect of RIME on ethnocultural empathy was more evident in male than female participants. 

A follow-up analysis revealed that males exhibited a statistically greater increase in empathy and its subdomains than females. This finding is intriguing given that previous research on cultural empathy has revealed no such gender differences or greater empathy shown by females than by males [13,23]. A closer look at the baseline measures for empathic awareness revealed that males were significantly lower on empathic awareness than females to begin with. That is, there was greater room for improvement in empathic awareness for males than females. Although this study enriches the current literature on empathy, further enquiry is needed to investigate the role that gender has on embodied learning and empathy.

Second, tolerance towards people from different cultural backgrounds was significantly increased following the RIME intervention. Although the baseline across the groups was relatively high before RIME and the sense of security was not different between learning groups, the significant increase after RIME suggests the intervention was effective in promoting the acceptance and interaction with those of other cultural backgrounds (see also, [12]). Taken together, these findings provide strong evidence that RIME has *culture-general* effects; that is, learning a musical instrument from another culture enhanced empathy and tolerance to other cultures in general. 

Third, social connectedness towards Chinese and Middle Eastern people significantly improved within each respective learning group after the RIME intervention. For example, participants who learned the Chinese *pipa* reported a significant increase in social connectedness towards Chinese people but not Middle Eastern people, whereas participants who learned the Middle Eastern *oud* reported a significant increase in social connectedness towards Middle Eastern people but not Chinese people. These results suggest that RIME may also have *culture-specific* effects. 

Why should music engagement elicit feelings of social connection? One reason is that feelings of social connection may result from the sheer enjoyment of culture-specific musicking. Indeed, in the open-ended responses, participants frequently cited enjoyment as a key experiential outcome from the RIME intervention. A second explanation is that exposure to and experience with active movement involved in learning and playing the *pipa* or *oud* activates a process of *social entrainment* that closes the “self–other gap” and increases social bonding with the specific cultural group [24]. Such an interpretation is consistent with research showing that the enjoyment of familiar culture-specific music is associated with feelings of interpersonal connectedness [25]. 

Fourth, a more favourable attitude towards ethnocultural groups was instilled, but this result was specific to particular cultural learning groups. Participants in the Middle Eastern *oud* group exhibited a significant increase in positive attitudes towards Middle Eastern people, whereas explicit attitudes towards Chinese people in the Chinese *pipa* group increased numerically but the changes were not statistically significant. Why were White Australians in the Middle Eastern *oud* group affected more by the intervention than those in the Chinese *pipa* group? Examination of baseline (pre-RIME) scores suggests attitudes against Middle Eastern people were more negative than attitudes towards Chinese (or Australian) people. As such, there was greater scope for an increase in positive attitudes towards Middle Eastern people than towards the other two ethnic groups. By engaging in embodied learning of the Middle Eastern culture, participants may have become more aware of their biases against Muslim communities and were willing to adjust their attitudes as a result. In comparison, biases against Chinese people were subtle and hence less impacted by the RIME intervention. The attitudes of Australians towards Australians increased but not by a statistically significant amount. However, in general, White Australians held a more positive attitude about themselves, as shown by the higher baseline with both groups. This is reflective of ingroup favouritism, which exists without the inclination to dislike other cultural groups.

Fifth, the RIME intervention reduced White Australian participants’ implicit bias against ethnocultural groups, as measured by IAT *D*-score. Specifically, we observed significant implicit biases against Chinese people and Middle Eastern people before the RIME intervention, but for the Chinese *pipa* group, no bias was detected after RIME. RIME with a Chinese instrument reduced implicit biases against Chinese people, but RIME with a Middle Eastern instrument had no impact on minimising implicit biases against Middle Eastern people.

It is striking that a two-part, four-hour program focusing on RIME was an effective strategy of decreasing implicit ethnocultural bias against Chinese people, but not Middle Eastern people. This pattern of results suggests that the effectiveness of the RIME intervention depends on the nature of the cultural bias (i.e., explicit or implicit) [8,26]. In this study, the baseline of explicit attitudes towards Middle Eastern people was lower, the RIME intervention made participants aware and rectified their attitudes; therefore, explicit attitudes towards Middle Eastern people were significantly improved. Implicit bias toward Middle Eastern people diverged from that trend, and the strong implicit bias against Middle Eastern remained strong after the RIME. One explanation might be the prolonged exposure to stereotypical media (i.e., depicting Middle Eastern people as terrorists) instils fear and threat among White Australians [1]. Fear and perceptions of threat activate the implicit bias against Middle Eastern people [27]. 

Another explanation might be the impact of cultural distance on intergroup relationships. In particular, Australia and China may be more culturally similar than Australia and the Middle East, in part because of the physical proximity of Australia and China, but also because there is a larger contingent of Chinese immigrants than Middle Eastern immigrants within Australia. Furthermore, the divergence also highlights that attitudes (especially racial biases) are often multilayered and people only have the ability to report the most recent top layer of (explicit) attitudes, seemingly unaware that they suppress the older layers of (implicit) attitudes [28]. The dynamic interaction of conscious and unconscious awareness of attitudes is complex; therefore, explicit attitudes sometimes converge and sometimes diverge with implicit attitudes. Furthermore, this divergence of implicit and explicit attitudes may be caused by the fact that implicit and explicit tests measure different manifestations of attitudes, namely, conscious introspection versus cognitive speed (for details on alternative tests, see [28]). Nevertheless, bodily engagement in physical musicking may have nurtured an embodied awareness of cultural differences [29], which may have triggered strategies of either reducing or holding on to that bias. Future research is needed to investigate the impact of different types of tests and durations of the intervention, and its efficacy for people with different levels of musical skill and trait empathy. Furthermore, preexisting knowledge of, and engagement with, the target culture could be assessed. In addition, despite the consistent approach adopted by the two instructors, subtle differences in the teaching styles of instructors could be monitored closely to identify any potential impact of teaching styles or demeanour on the study’s results.

Lastly, qualitative data from the open-ended questions provided additional evidence in support of the hypotheses. For example, there was agreement across many participants that the study *helped them understand the music culture and feel more connected with the people of that culture*. Although the issue of prejudice and racism was not mentioned during the RIME intervention program, such qualitative reflections supported the quantitative findings that RIME increased intercultural empathy.

Overall, the findings of the present study provide the first empirical evidence that RIME is an effective strategy for enhancing ethnocultural understanding, social connectedness, and attitudes. The findings underscore the benefits of music learning in multicultural societies and suggest that embodied engagement with cultural traditions may be a crucial element in unlocking such benefits. This study also demonstrated that implicit biases are often persistent and may require greater durations of RIME for changes to be detected. Negative implicit biases are deeply rooted, which may explain why bias against Middle Eastern people was persistent despite our RIME intervention. Further longitudinal studies are needed where a RIME intervention is implemented over many months with multiple measures over time. This will shed light on whether RIME can reliably reduce implicit biases and the time-course of such effects. Furthermore, investigating the effects of learning in groups could provide further evidence for the efficacy of RIME intervention. Participants in the current study were highly educated Australian residents of predominantly Anglo origin living in Sydney. Future research could investigate the effects of RIME for other populations, such as people living in non-urban regions, or people of non-dominant cultural origins. It would also be interesting to compare the impact of RIME for groups of individuals with opposing political affiliations, who may have different baseline understandings or prejudices concerning other cultures. Future studies should also compare the experience of taking part in the study for different age groups. Such a comparison might provide insight into the efficacy of RIME for younger participants who may be constructing and developing their cultural identities and attitudes versus participants for whom such identities and attitudes are well established.

## 5. Conclusions

To conclude, training White Australians on the Chinese *pipa* or Middle Eastern *oud* can effectively nurture intercultural understanding. As predicted by the Intercultural Music Capacities Model, RIME successfully promoted intercultural understanding, leading to benefits such as increased ethnocultural empathy and tolerance, feelings of social connection, improved attitude, and reduced implicit bias. The findings provided strong support for the IMCM framework and suggest a causal impact of the intervention. Future research into the nature of implicit cultural bias, for example, implicit stereotypes and cultural bias hierarchy [30,31], may be useful for further refinement of the model. Our RIME intervention is short, effective, enjoyable, and has the potential to be of enormous value as an evidence-based educational program to nurture intercultural understanding in multicultural societies worldwide. 

## Figures and Tables

**Table 1:** An Example of the Sequence of Trial Blocks in IAT, White Australian versus Chinese.

Block	Number of Trials	Items Assigned to “E” Key	Items Assigned to “I” Key
B1	20	Australian faces	Chinese faces
B2	20	Positive words	Negative words
B3	20	Australian faces + positive words	Chinese faces + negative words
B4	40	Australian faces + positive words	Chinese faces + negative words
B5	20	Chinese faces	Australian faces
B6	20	Chinese faces + positive words	Australian faces + negative words
B7	40	Chinese faces + positive words	Australian faces + negative words

Note. Australian faces refer to six grey-scaled Australian face images, three male and three female; Chinese faces refers to six grey-scaled Chinese face images, three male and three female; positive words refer to eight positive adjectives, “Friendly, Glorious, Happy, Joyous, Lovely, Magnificent, Pleasant, Wonderful”; negative words refer to eight negative adjectives, “Angry, Awful, Disgust, Evil, Failure, Horrible, Nasty, Terrible”.

**Table 2:** Means and Standard Deviations for Feedback Questions.

Question	*M* (*SD*)
1. It challenged me.	4.29 (0.56)
2. It gave me a sense of achievement.	4.76 (0.44)
3. It helped me understand [Field-Ethnicity] ^1^ culture.	4.15 (0.79)
4. It made me feel more connected to [Field-Ethnicity] people.	4.05 (0.81)
5. It made me feel more connected with others (all people in the world).	3.93 (0.82)

Note. ^1^ [Field-Ethnicity] refers to the actual text displayed, “Chinese” or “Middle Eastern”.

## Data Availability

The data presented in this study are available upon request from the corresponding author.
